# Correlation between Indoor Environmental Data and Biometric Parameters for the Impact Assessment of a Living Wall in a ZEB Lab

**DOI:** 10.3390/s20092523

**Published:** 2020-04-29

**Authors:** Francesco Salamone, Benedetta Barozzi, Ludovico Danza, Matteo Ghellere, Italo Meroni

**Affiliations:** Construction Technologies Institute, National Research Council of Italy (ITC-CNR), Via Lombardia, 49, 20098 San Giuliano Milanese, (M.I.), Italy; barozzi@itc.cnr.it (B.B.); danza@itc.cnr.it (L.D.); ghellere@itc.cnr.it (M.G.); meroni@itc.cnr.it (I.M.)

**Keywords:** living wall, wearable, IoT, machine learning

## Abstract

Users’ satisfaction in indoor spaces plays a key role in building design. In recent years, scientific research has focused more and more on the effects produced by the presence of greenery solutions in indoor environments. In this study, the Internet of Things (IoT) concept is used to define an effective solution to monitor indoor environmental parameters, along with the biometric data of users involved in an experimental campaign conducted in a Zero Energy Building laboratory where a living wall has been installed. The growing interest in the key theory of the IoT allows for the development of promising frameworks used to create datasets usually managed with Machine Learning (ML) approaches. Following this tendency, the dataset derived by the proposed infield research has been managed with different ML algorithms in order to identify the most suitable model and influential variables, among the environmental and biometric ones, that can be used to identify the plant configuration. The obtained results highlight how the eXtreme Gradient Boosting (XGBoost)-based model can obtain the best average accuracy score to predict the plant configuration considering both a selection of environmental parameters and biometric data as input values. Moreover, the XGBoost model has been used to identify the users with the highest accuracy considering a combination of picked biometric and environmental features. Finally, a new Green View Factor index has been introduced to characterize how greenery has an impact on the indoor space and it can be used to compare different studies where green elements have been used.

## 1. Introduction

Users’ satisfaction in indoor spaces is a key point in the design process of a comfortable building environment. Different technical solutions to be applied to the envelope and thermal plant systems have been developed, studied and diffused for commercial purposes. The study of the effects produced by the presence of greenery solutions in indoor environments has engaged the international scientific literature since the late 1980s on some different and complementary fronts, leading to a significant spread of green potted elements and of a vertical green façade, known as a “vertical garden” or “living wall”.

The scientific research has focused, for example, on the analysis of the micro-environmental fallout with regard to the ability of specific plants to contribute to the improvement in Indoor Air Quality (IAQ) through the abatement of indoor air pollutants. Wolverton’s studies [1] have shown, for example, that low-light indoor plants, associated with soil microorganisms and combined with active carbon filters, have a strong potential to improve IAQ by removing organic tracks of air pollutants in energy-efficient buildings (the most exposed to the problems of sick environment). Moving from the studies carried out by NASA, most recently, Pegas et al. [2] corroborated the previous results concerning the ability of plants to improve IAQ, reducing air pollutants’ (CO_2_, VOC_S_ and PM10) concentrations.

Other studies focused their attention on the potential of specific ornamental potted plant in removing VOCs from indoor air, concluding that greenery removal efficiency is strictly influenced by aspects such as plant species, light intensity, indoor temperature, VOCs concentration and identity [3], or, in other cases, by the microorganisms closely associated with the used growing medium and the root system [4]. Irga et al. [5] studied the removal potential of CO_2_ and VOCs from indoor environments comparing a conventional potting mix and hydroculture, whereas Darlington et al. [6] based their studies on the use of a biofiltration system, composed of a series of bioscrubbers, through which the air of the room, a hydroponic growing region, has been sucked. Whatever approach is tested, all the research mentioned clearly indicates that the removal of indoor air pollutants is possible.

Some other researchers have focused their scientific interests on the active contribution of greenery systems to influence some indoor parameters such as temperatures and relative humidity.

Gunawardena and Steemers [7], in their bibliographic review concerning the outdoor and indoor applications of “vertical green systems” underline how indoor living walls are a very recent innovation. Consequently, the effects of using a living wall on the indoor environment are still poorly assessed.

Only a few studies have been carried out on the real effects of living walls on indoor environment frequented by humans. Fernàndez-Canero et al. [8], for example, investigated the impact of a living wall on indoor temperatures and relative humidity installed in a hall inside a section of the University of Seville (Spain): the results quantified the summer cooling effect with an average reduction of 4 °C, over the room temperature, and registered a significant increase in the relative humidity level of the air both near the living wall and in the overall hall room. A subsequent work carried out by the same team of researchers [9], investigated the effects on the indoor temperature and relative humidity of an active living wall, in other words, a system in which air is forced to pass through the living wall to take advantage of its evaporative cooling potential [8], reducing the ventilation requirements of the room. However, the literature is still insufficient and must be deepened, going beyond the analysis of the relationship between the presence of the living wall and indoor environmental parameters, through an all-encompassing analysis that considers environmental and biometric parameters and possible correlations with the presence, for example, of a living wall.

The remaining literature analyses the energy-environmental effects of a living wall, generally applied on an outdoor environment. Mazzali et al. [10], for example, realized three living wall field tests to investigate their potential effects on the energy behavior of the building envelope, monitoring both the external surface with respect to a bare wall, and the incoming/outgoing heat flux. More recently, a study carried out in Australia [11] was focused on the monitoring of relative humidity and temperatures comparing an outdoor living wall with a bare wall, studying the effects on both the surrounding microclimate and the indoor back wall. Many other studies have been carried out in this direction, always considering the outdoor installation of living walls.

Finally, other researchers have focused their studies on the analysis and verification of the psycho-physiological response of users to the presence of real or simulated (through virtual reality or photos) potted flowering and foliage plants: the early scientific studies, carried out between the late 1980s and the beginning of the new Millennium, demonstrated that human–plant interaction ensures a physiological reduction in stress in a very quick lapse of time, almost within minutes of exposure [12,13,14], recording an improvement in psychological [15,16], emotional [17] and cognitive health [18,19].

The biometric effects due to the use of this solution are quantified in few cases and in different indoor environments (hospital rooms, offices, schools).

Chang and Chen [20] describes the effects of different window views and indoor plants on the human psychophysiological response of 38 volunteers in a laboratory equipped as an office, considering six different combinations of window views and indoor plotted plants. The results conducted considering electroencephalography, electromyography and blood volume pulse have shown that the window view has a greater effect on the state of anxiety when compared with indoor plants.

Dijkstra et al. [21] reports the result of an infield investigation regarding the possibility of using natural elements to reduce the stress in a hospital room considering a sample of 77 volunteers with no direct acquisition of biometric parameters. The results show that the perceived stress of patients is reduced in the presence of indoor plants. The same environment was considered by S. Park et al. in [22], where they studied the therapeutic influence of plants on a sample of 90 patients through the acquisition of systolic and diastolic blood pressure, body temperature, heart rate and respiratory rate.

In [23], the psychological relaxing effects due to the exposure to rose flowers in a conference room occupied by 31 males, were reported, while in [24] the shared feeling of greater comfort and relaxation of 85 students was determined when exposed to the vision of a *dracaena* plant. Choi et al., in [25], introduced an index of greenness in indoor space in terms of preferred level of greenery considering an equipped room in a university laboratory, where 103 volunteers took part in the test. A. E. van den Berg et al. in [26] evaluated the restorative impact of living walls in different classrooms of elementary schools. J. Yin et al. [27] performed cognitive tests on a sample of 28 volunteers.

The present paper differs from the above mentioned because it considers the correlation analysis among monitored environmental variables and biometric parameters in a research campaign carried out considering nine different users who alternatively occupied a ZEB lab room [28] equipped as a working station with four different system configurations. The article intends to investigate the complex interaction among the environment, occupants and the presence of a living wall in order to define new models that fill the gap of the current methodologies to design comfortable, usable, adaptable and energy-efficient buildings, emphasizing the potential of Internet of Things (IoT) and Machine Learning (ML) techniques.

Table 1 reports the most important features of the proposed study if compared with the reference literature reported in the introduction, which have provided for the involvement of participants in real-life contexts.

The paper is structured as follows: the second chapter describes the experimental set-up used to define the dataset. The third chapter reports and discusses the outcomes of the dataset analysis with the machine learning (ML) techniques. Finally, a conclusion about the implication of the proposed framework within building design and future development is reported in the last chapters.

## 2. Experimental Set-Up

The experimental set-up used to define the dataset managed with ML models for correlation analysis consists of a laboratory equipped with a set of sensors used for the acquisition of environmental data and wearable devices were used for the monitoring of biometric parameters of the users involved in the test. Meanwhile, a Google Form is used to record personal comfort perception. During four consecutive weeks, four different configurations are considered as described in detail in Section 2.2.

### 2.1. Test Case, Monitoring System and Questionnaire

This study is the first part of a wider experimentation aimed at analysing the behaviour of an indoor living wall from different point of views (hygro-thermal, acoustic, air quality) in a room that simulates an office over a period of one year, in adaptation to different indoor hygro-thermal conditions, in both active and passive plant conditions. Specifically, it aims to analyse the impact of the indoor living wall in terms of the variation in the individual biometric response of occupants during the month of May 2019.

**Figure 1:**
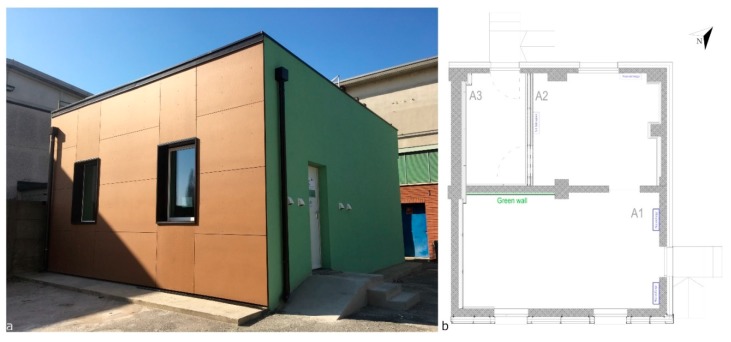
LabZEB: (**a**) outdoor view; (**b**) indoor plan.

The experimentation is carried out within the CNR-ITC ZEB Laboratory in San Giuliano Milanese near Milan, in the A1 room (Figure 1a). It has dimensions of about 640 × 370 × 295 (L × W × H, expressed in cm) with two windows of 110 × 160 (L × H in cm) facing a south-east orientation with white plastered walls, white panels for the suspended ceiling and metal black tiles for the floor. The living wall, provided by the company Sundar Italia, occupies the north-west side of the A1 room (in green in Figure 1b) with an area of about 7 m^2^. It consists of an indoor living wall mounted on an aluminum frame, on which special panels are installed and connected each other. The frame is fixed to the support wall (A1 room, Figure 1b) with a special system that facilitates the natural renewal of the air and does not damage the wall. Panels are realized in pvc and covered by three layers of felt: plants are rooted in those felt layers and grown in hydroponics in the absence of a growing medium.

Plants were selected directly by Sundar Italia depending on the indoor environmental conditions to which they would be exposed. The chosen species are two variants of *ficus repens* (or *ficus pumila*) characterized by green or white edged leaves. An artificial illumination system was installed in order to guarantee that plants get the right amount of light and the correct light spectrum they need for their grow and conservation. The drip irrigation system, positioned on the top frame of the living, is completely autonomous and automatic.

Two Heat Recovery systems (HR) are installed on the north-east wall of the room (in blue in Figure 1b) consisting of decentralized ventilation units with heat recovery designed for installation in residential and commercial spaces. Considering that, for the Italian climatic zone E [29] in which the experimentation took place, the month of May presents the optimal climatic conditions for tests in passive conditions, the heating and cooling plants were switched off. Therefore, the HR in this phase worked exclusively as an indoor air renewal system.

Within A1, two complete workstations (desk, chair, PCs) are installed, facing a south-west direction. Each workstation is completed by a monitoring system consisting of a thermo-hygrometric sensor for the measurement of air temperature and relative humidity, a black globe thermometer and a hot wire anemometer for the measurement of both air velocity and temperature. In addition, occupants wear a smart device for biometric data acquisition.

**Figure 2:**
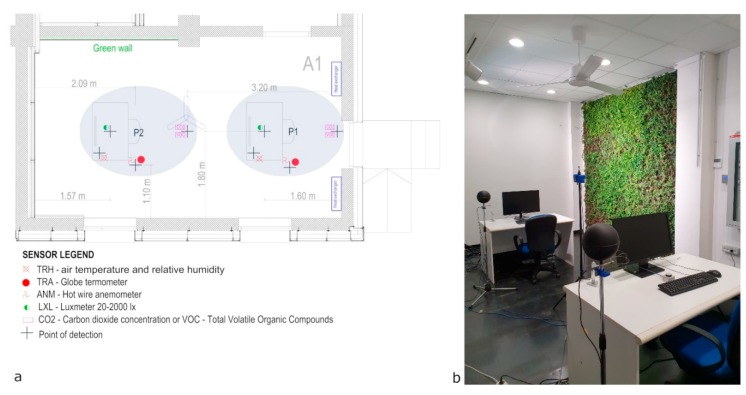
A1 room: (**a**) spatial distribution of sensors; (**b**) photo of the room set up.

Figure 2 reports the spatial distribution of the sensors used for the monitoring of indoor environmental variables.

Table 2 reports the metrological characteristics of the environmental and biometric sensors.

While it is clear why the above environmental sensors are considered, it is not clear which of the above-mentioned biometric parameters could be affected by the surrounding environment. For this reason, all data recorded by the wearable device are considered. The experimentation, where participants perform common office tasks (typing and reading with or without video terminal support), is structured in two sections: one in the morning (3-h long) and one in the afternoon (2-h long). The sessions are separated by an interval time of one hour in order to nullify/minimize the effects of the prolonged occupants’ presence in relation to indoor environment conditions and the fatigue of participants. At the end of each section, before leaving the room, the user involved in the test answers some questions using a web-based Google Form (Table 3). All answers are based on a five-point Likert scale, used to allow the individuals to express how satisfied or unsatisfied they are with a particular comfort condition.

As the impact of several factors on the overall categorization of the Indoor Environmental Quality (IEQ) is unclear and further research is needed considering the numerous differences in terms of “characteristics of occupancy, ventilation type, office type, etc.” [30], it was decided, starting from the answers to the questions on thermo-hygrometric perception (Q2 in Table 3), air quality (Q3) and lighting quality (Q4), to derive a simple IEQ score, defined as the weighted average of the three values. The data derived from the participants’ feedback are considered in the definition of the dataset, but they are not considered in the ML approach.

### 2.2. Configurations

The monitoring campaign lasted for four consecutive weeks in May, each of which is characterized by one of the below four configurations (Table 4) defined considering a different combination of possible settings.

The choice of four consecutive monitoring weeks during the month of May (spring season) is due to the following reasons:

- Avoid a high external temperature difference between configurations, keeping the possibility of comparing results;
- Ensure acceptable indoor temperature levels for the researchers involved in the experiment, as the cooling system was switched off (passive conditions); in the hottest periods of the year (summer months), the indoor temperatures should require the use of cooling plants;
- Ensure the minimum indoor temperatures required by ficus repens: for their survival, a minimum temperature of 10 °C is required, which is hard to maintain in the coldest periods of the year without the use of HVAC plants;
- Ensure that the indoor humidity doesn’t exceed the maximum level tolerated by the selected green essences without risking lowering the indoor temperature too much; using the HR (configuration 2 and 4) to mix the air and to mitigate the humidity level created both by the irrigation of the living wall and by the evapotranspiration of the plants in colder periods would have led to an excessive drop in perceived indoor temperatures.

**Figure 3:**
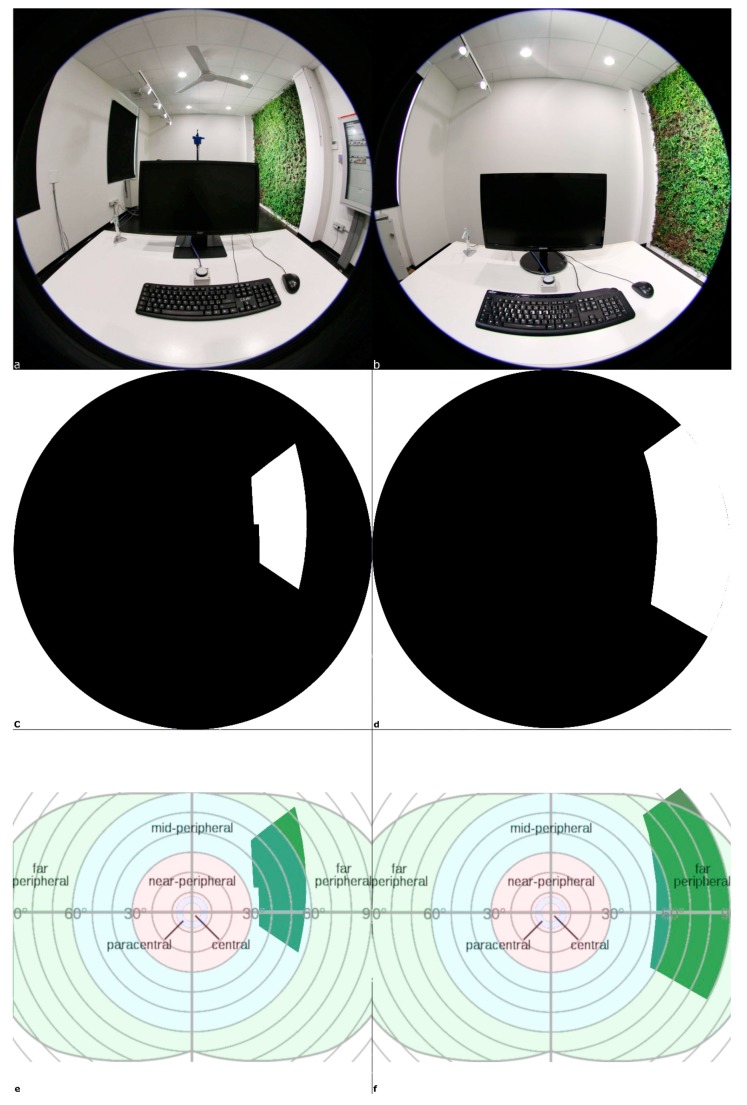
P1 and P2 Field of View: (**a**) P1 photo; (**b**) P2 photo; (**c**) P1 Black and white file for Green View Factor (GVF) calculation; (**d**) P2 Black and white file for GVF calculation; (**e**) P1 green area peripheral vision; (**f**) P2 green area peripheral vision.

The research campaign is carried out considering nine users who paired occupied the two workstations (P1 and P2 in Figure 2a) available in the A1 lab room. The field of view of workstations P1 and P2 is reported in Figure 3a,b, respectively. Figure 3c,d is used to define, in a kind of parallelism with the Sky View Factor (SVF) estimation, using a fisheye [31], the Green View Factor (GVF), an index introduced to indicate the fraction of green area on the surface of a hemisphere centered on the point of analysis (the upper edge of the monitor, in the middle position). The GVF for P1 is 0.061, while for P2 it is 0.114. Very low values indicate that a small portion of the space centered at the analysis point is occupied by the green area. In addition, the very similar values indicate that the green areas resulting from the different perspective view for the two positions are roughly equivalent. Figure 3e,f highlights how the green areas occupy the peripheral visions [32] of the Field of View: in P1, it covers the right far-peripheral area, while in P2 it covers the right part of mid-peripheral vision.

The workstation orientation and placing within the room emphasize particular indoor discomfort effects due, for example, to the presence of the two south-east exposed windows in proximity of P1 and P2 that could favour daylight discomfort or radiant asymmetry.

### 2.3. Dataset Attributes

**Figure 4:**
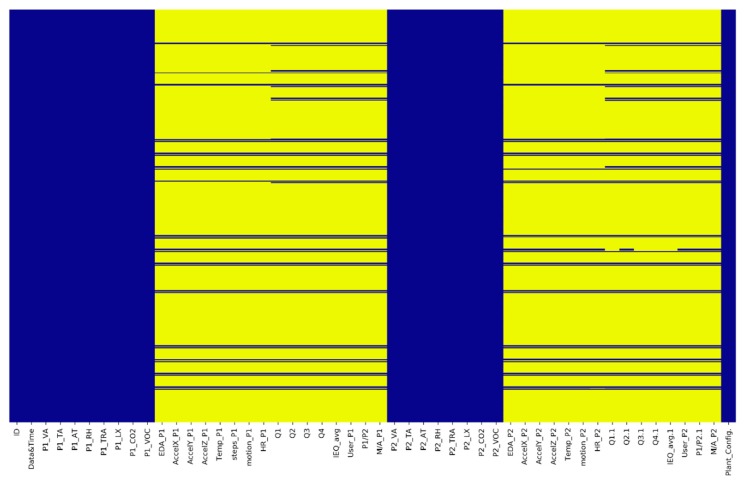
Starting dataset and related parameters: in yellow—null data, in blue—non-null data.

The experimental approach described above defined a preliminary dataset structured considering all environmental, biometric and user feedback data consisting of a total of 43,100 instances and 50 attributes. The heat map shown in Figure 4 can verify the consistency of the defined dataset.

It is possible to highlight how this is completely imbalanced. The following chapter describes how it has been filtered and used in the ML approach.

The above-mentioned set-up and dataset try to answer to the following questions:

- What are the main environmental variables and models useful to accurately classify the adopted plant configurations?
- Are the biometric data useful to classify the adopted plant configurations? If so, which features are the most important?
- How does combining environmental and biometric data affect the accuracy of the model?

## 3. Results and Discussion

### 3.1. Data Filtering and Dataset Structure

**Figure 5:**
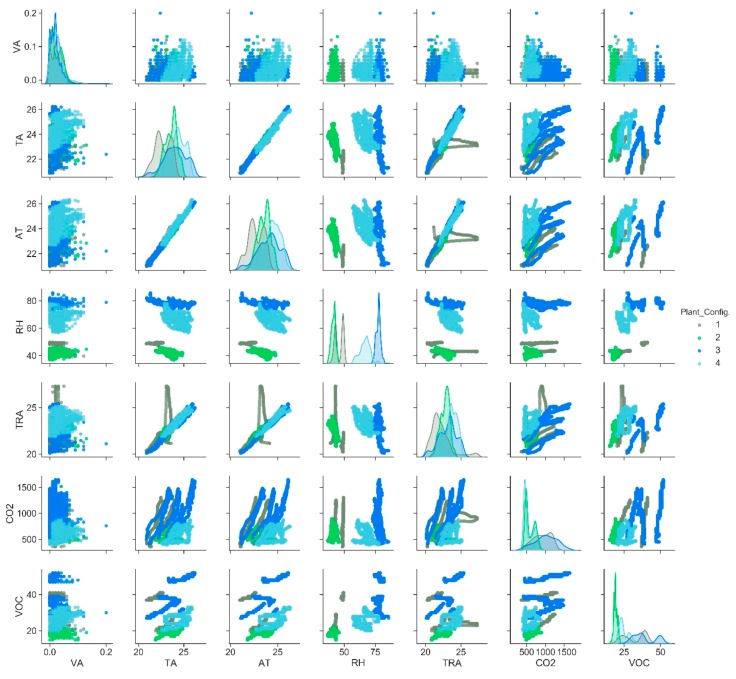
Correlation matrix between environmental parameters.

To overcome the limitations of the starting imbalanced dataset due to a small number of available data, a new one (Figure 5) is defined, starting from the above situation, by applying the following steps:

- Filtering Not Available Number (NaN) by using DataFrame.dropna pandas function [33] considering a subset defined starting from a list of specific columns (subset= [“filtered_eda_P1”, “HR_P1”, “filtered_eda_P2”, “HR_P2”, “Q1.1”]);
- Defining the P1 subset of data, by using DataFrame.loc pandas function [34], to consider only the P1 label in the P1/P2 column and DataFrame.rename function [35], renaming specific attributes (columns={‘P1_VA’: ‘VA’, ‘P1_TA’: ‘TA’, ‘P1_AT: ‘AT’, ‘P1_RH’: ‘RH’, ‘P1_TRA’: ‘TRA’, ‘P1_LX’: ‘LX’, ‘P1_CO2’: ‘CO2’, ‘P1_VOC’: ‘VOC’, ‘EDA_P1’: ‘EDA’, “AccelX_P1”:”AccelX”, “AccelY_P1”:”AccelY”, “AccelZ_P1”:”AccelZ”, “Temp_P1”:”Temp”, “motion_P1”:”motion”, “HR_P1”:”HR”, “User_P1”: “User”, “M/A_P1” : “M/A”});
- Defining the P2 subset of data, by using DataFrame.loc pandas function [34], to consider only the P2 label in the P1/P2 column and DataFrame.rename function [35] renaming specific attributes (columns={‘H_VA’: ‘P2_VA’, ‘H_TA’: ‘P2_TA’, ‘P2_AT’: ‘AT’,’P2_RH’: ‘RH’, ‘P2_TRA’: ‘TRA’, ‘P2_LX’: ‘LX’, ‘P2_CO2’: ‘CO2’, ‘P2_VOC’: ‘VOC’, ‘EDA_P2’: ‘EDA’, “AccelX_P2”:”AccelX”, “AccelY_P2”:”AccelY”, “AccelZ_P2”:”AccelZ”, “Temp_P2”:”Temp”, “motion_P2”:”motion”, “HR_P2”:”HR”, “Q1.1”:”Q1”,”Q2.1”:”Q2”, “Q3.1”:”Q3”, “Q4.1”:”Q4”, “Q5.1”:”Q5”, “IEQ_avg.1”:”IEQ_avg”, “User_P2”: “User”, “P1/P2.1”:”P1/P2”, “M/A_P2” : “M/A”});
- Using the DataFrame.join pandas function [36] to merge P1 and P2 subset data.

The derived dataset is characterized by a series of 5692 instances and 25 attributes, each of which is defined in Table 5.

### 3.2. ML Approach

The defined dataset is used through the ML approach to identify the correlation between some variables and different target values.

#### 3.2.1. Environmental Parameters Correlation Considering the Plant Configuration as a Target Value

The correlation between environmental monitored data and the four considered configurations is reported in the scatter matrix plot of Figure 5.

**Figure 6:**
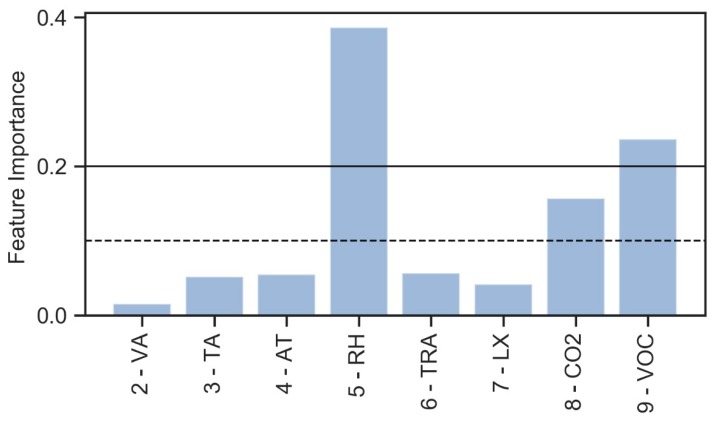
Feature importance—environmental data for plant configuration identification.

Figure 6 highlights how some attributes (RH, the pair RH–VOC) are useful to predict the plant configurations, each characterized by a specific color as reported in the legend. However, it is not possible to identify which attributes would be the best to validate and predict the plant configuration based on this set of environmental data. For this purpose, an Extremely Randomized Tree technique [39] with Python’s scikit-learn tool [40] is considered, thus allowing to verify the importance of environmental features to identify the categorical target label (Figure 6).

Defined two threshold feature importance values equal to 0.1 and 0.2, it is possible to identify the most important variables that can be used to determine the plant configuration (Figure 6).

The RH has a predominant impact when compared with all other environmental data. Considering the two threshold values defined previously, the following lists of variables are considered:

- [5—RH, 8—CO2, 9—VOC];
- [5—RH, 9—VOC].

An analysis is then carried out to identify, according to the ML approach, which the algorithm can identify, with the highest accuracy, the plant configuration adopted. For this purpose, a set of different algorithms are considered: Logistic Regression (LR) [41], Linear Discriminant Analysis (LDA) [42], K-Nearest Neighbors (kNN) [43], Classification and Regression Trees (CART) [44], Extra Tree Classifier (ETC) [40], Gaussian Naïve Bayes (NB) [45], Support Vector Machines (SVM) [46], Random Forest (RF) [47], eXtreme Gradient Boosting (XGBoost) [48].

Each of the considered algorithms is characterized by a different solving approach. Thus, for example, LR is a supervised classification method usually used when the target variable is categorical. LDA is a supervised technique used to reduce the number of dimensions (i.e., variables) in a dataset while retaining as much information as possible. kNN is a supervised learning algorithm that considers different centroids and uses a Euclidean function to compare and classify each point to the group to optimize it to place with all closest points to it. CART is referred to as “decision trees” because it takes an instance, traverses the tree, and compares important features with a determined conditional statement. Whether it descends to the left lower branch or the right depends on the result. ETC is a type of ensemble learning technique which aggregates the results of multiple de-correlated decision trees collected in a “forest” to output its classification result. In concept, it is very similar to an RF and only differs from it in the manner of construction of the decision trees in the forest. NB is based on Bayes’ theorem that assumes independence between predictors. A Naïve Bayes classifier will assume that a feature in a class is unrelated to any other. In particular, the selected NB model, implements the Gaussian Naïve Bayes algorithm for classification. SVM is a supervised classification algorithm that plots a line that divides different categories of your data and optimizes it to ensure that the closest points in each group lie farthest from each other. Finally, XGBoost, follows the principle of gradient boosting, and currently it is considered to be one of the most useful libraries to build accurate models on structured data.

The dataset is divided into two subsets, composed of 80% and 20% of values. The former is used to train the models and the latter for the test.

The “accuracy” metrics [49] have been used to evaluate the different algorithms, which in this specific contest is defined as the ratio between the correct number of instances predicted, divided by the total number. A k-fold cross validation [50] equal to 10 has been considered. Below is the average value for each algorithm.

Table 6 shows the average accuracy and the standard deviation for the different considered algorithms.

As reported in [51], tree-based models always work better than the alternatives when there is no hyperparameter tuning. To verify this circumstance, the tuning of the hyperparameters was carried out for LR, KNN, CART, ETC, SVM, RF and XGBoost. The values with an asterisk in Table 6 are those obtained in the tuning of hyperparameters: LDA and NB have not been considered because they have no hyperparameter to tune [42,52].

Table 7 shows the hyperparameters tuned and their corresponding ranges.

The statistical significance of the results is verified using the ANalysis Of VAriance (ANOVA) test SciPy function [53]; data of each sample are normally distributed and with the same standard deviations, because nine data samples are considered, one for each considered model, consisting into the array of 10 accuracies. The p-values are lower than 0.05, demonstrating the statistical significance of the results.

The XGBoost and RF with three features have the same highest average accuracy and lowest values of standard deviation. Considering only two features, the XGBoost records the best results. Consequently, the validation values are defined (Table 8) in terms of:

- *Precision* defined as a measure of a classifiers exactness;
- *Recall* considered as the completeness of the classifier;
- *f1-score*, a weighted average of precision and recall;
- *Support*, the number of occurrences of each label in y true.

#### 3.2.2. Biometric Parameters Correlation Considering the Plant Configuration as a Target Value

**Figure 7:**
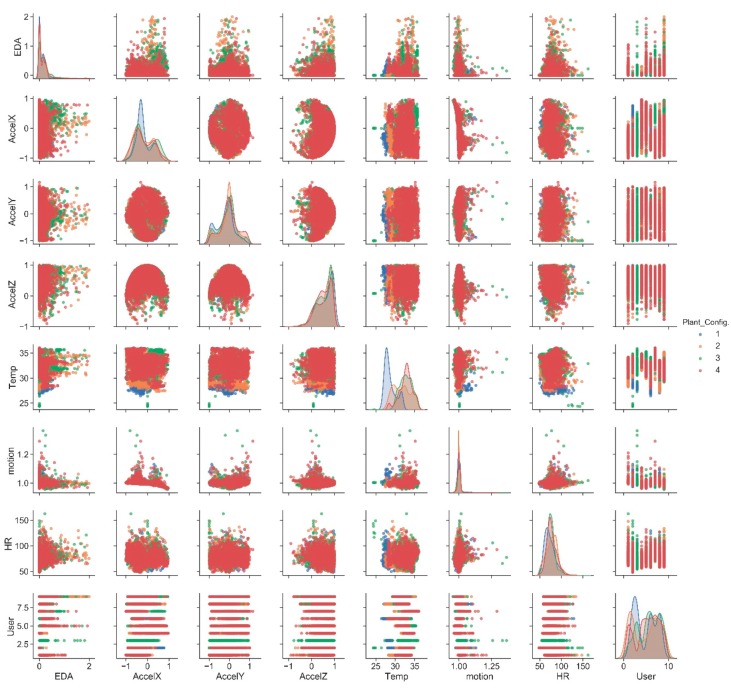
Correlation matrix between biometric parameters.

The correlation between biometric variables and the four considered configurations is investigated (Figure 7).

**Figure 8:**
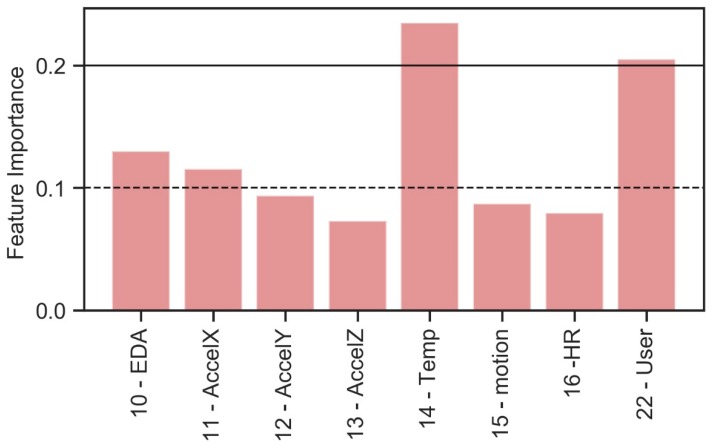
Feature importance—biometric parameters for plant configuration identification.

In this case, it is not possible to identify which algorithms would be the best to validate and predict the plant configuration based on this set of biometric data. Considering the same ML approach used in the previous case, an analysis is then carried out to identify the relationship among all available biometric data parameters and the plant configuration. First, the sub-dataset is analyzed in order to identify the importance of individual features to identify the categorical label “plant configuration” (Figure 8).

Defined the same threshold feature importance values equal to 0.1 and 0.2, the following biometric parameters are selected for the identification of plant configuration:

- [10—EDA, 11—AccelX, 14—Temp, 22—User];
- [14—Temp, 22—User].

Table 9 shows the average accuracy and the st.dev. for the considered algorithms.

The values with an asterisk in Table 9 are those obtained considering the tuning of hyperparameters considering the same parameters and ranges reported in the previous Table 7.

XGBoost algorithm maintains the best level of average accuracy and lowest standard deviation considering the two lists of features. The validation values (Table 10) in terms of precision, recall, f1-score and support confirm the good results of the selected algorithm.

#### 3.2.3. Selected Biometric and Environmental Parameters Correlation Considering the Categorical Label User as a Target Value

**Figure 9:**
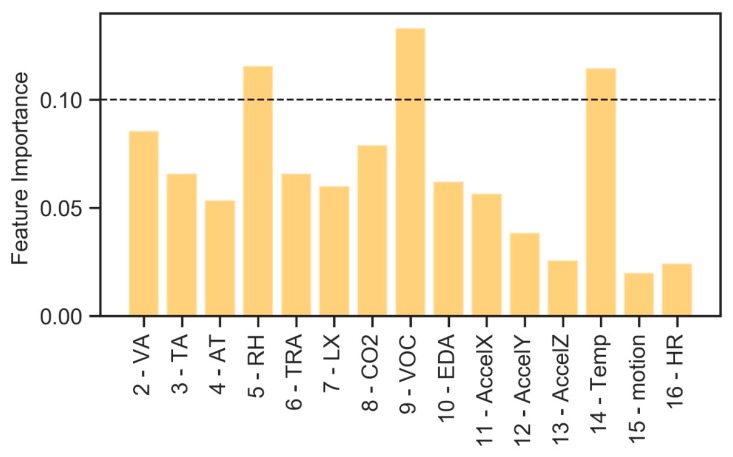
Feature importance—environmental and biometric data for user identification.

In the Section 3.2.2, two lists of biometric features are selected. In both, there is the User feature. It is possible to replace the categorical label *22—User* following the same approach, considering all environmental and biometric data. In this way, it is possible to verify the importance of individual features to identify the target feature, Users (Figure 9) thus highlighting the interconnection among environmental parameters and biometric data, as discussed in recent studies [54,55].

Table 11 shows the average accuracy and the standard deviation for the considered algorithms and selected features.

The validation values (Table 12) in terms of precision, recall, f1-score and support confirm the good results of the XGBoost algorithm.

#### 3.2.4. Selected Biometric and Environmental Parameters Correlation Considering the Plant Configuration as a Target Value

For the definition of the plant configuration, it is therefore possible to use a restricted set of environmental data (5—RH, 8—CO2, 9—VOC or 5—RH, 9—VOC) or biometric (10—EDA, 11—AccelX, 14—Temp, 22—User or 14—Temp, 22—User). The possibility of replacing the categorical label “User” with a selection of biometric and environmental data (5—RH, 9—VOC, 14—Temp) has been verified. Ultimately, therefore, the two lists reported below are considered to evaluate the goodness of the models in defining the target values *Plant.Config.*:

- [5—RH, 8—CO2, 9—VOC, 10—EDA, 11—AccelX, 14—Temp];
- [5—RH, 9—VOC, 14—Temp].

Table 13 shows the average accuracy and the standard deviation for the different considered algorithms.

The validation values (Table 14) in terms of precision, recall, f1-score and support confirm the good results of the XGBoost algorithm.

#### 3.2.5. Model Interpretability 

Summarizing from the results of the previous paragraphs, it is possible to highlight how, in this specific case, for the prediction of plant configuration, a maximum of three environmental parameters are useful (*5—RH, 8—CO2, 9—VOC),* while considering the environmental parameters, it is possible to consider four features (*10—EDA, 11—AccelX, 14—Temp, 22—User*). In this study, it has been possible to highlight how the *User* feature can be identified considering a mixture of environmental and biometric parameters (*5—RH, 9—VOC, 14—Temp*). Then, it has been possible to mix the selected environmental and biometric data to define an overall performance in defining the plant configuration. This has been achieved considering only the three environmental features or the mix of six features (environmental and biometric); the results are quite comparable considering the XGBoost-based model.

**Figure 10:**
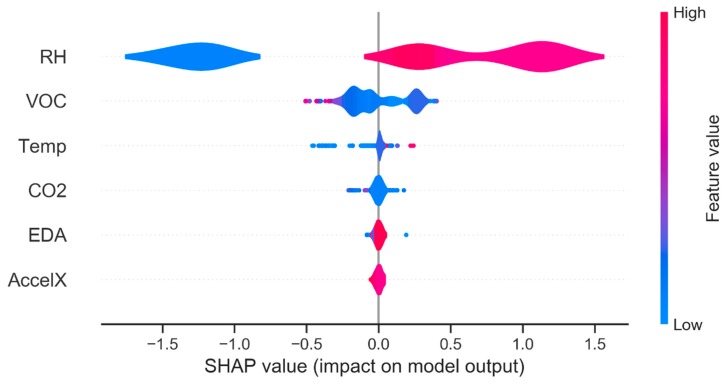
SHapley Additive exPlanations (SHAP) value plot.

For a long time, models have focused on reaching high performances without verifying or, better, explaining the causes of these results and their sense. In this specific case, in order to verify if the adoption of this set of selected environmental and biometric features is relevant, the SHapley Additive exPlanations (SHAP) library was used. This is a game theoretic approach that allows explaining the output of any machine learning model [56,57,58]. The set of six features is considered (*5—RH, 8—CO2, 9—VOC, 10—EDA, 11—AccelX, 14—Temp*) because it can obtain the highest precision for all the four plant configurations. The SHAP value plot (Figure 10) shows the distribution of the impacts of each feature on the model output.

Figure 10 summarizes the following useful information:

- Variables are classified in descending order of importance;
- The horizontal location shows whether the effect of that value is associated with a positive or negative impact on the prediction of target feature;
- Colour shows the feature value: high is in red and low in blue.

This reveals, for example, that a high *RH* has a positive impact on the quality rating. The “high” comes from the prevalent red colour, and the “positive” impact is shown on the X-axis. Similarly, the low values of the *VOC* feature have an impact on the model prediction comprised between −0.5 and 0.5.

The Figure 10 demonstrates that the *RH* feature has the dominant effect among the selected environmental and biometric data, while the *VOC*, *Temp* and *CO2* features could have a limited impact in defining the Plant configuration, where *Temp* is more important than the environmental variable *CO2*. The effect of the other variables (*AccelX* and *EDA*) is almost obscured by the dominant weight of all other considered features.

### 3.3. Discussion

The present research draws its foundation from the increased impact of IoT solutions, showing how different plant configurations also based on the adoption of greenery, could affect not only the environmental indoor parameters as expected, but also the biometric parameters of users that occupy the indoor environment. The use of IoT system has introduced new approaches of assessment of IEQ, allowing revising classical standard approaches. De facto, the approaches reported in the references have shown that the different evaluation methods which have been introduced follow independent and unrelated strands to assess the influence of the presence of a living wall in indoor environments, e.g., according to the psycho-physiological response of the users or the variation in indoor environmental parameters. On the contrary, the experimental bibliographic evidence has shown that indoor living walls have the ability to influence environmental, psychological and physiological aspects, if appropriately sized and calibrated. The use of supervised machine learning approaches allows recognizing correlations among different features of a conspicuous dataset. These are the reasons that led to proposing this methodological approach as the new horizon of evaluation of the effects produced by living walls in different indoor environments. The most important aspect of machine learning is repetitiveness, because the more the models are exposed to data, the more they are able to adapt independently. In this context, it could be useful to share a useful dataset structure that allows ML to learn from previous processing and to produce results or make decisions, for example, in the context of Building Automation, that are reliable and applicable to different contexts. ML models allow to understand the humans’ sentiments through automated systems, thus allowing to give the accurate answer to daily questions about the management and control of the building system as a whole.

Focusing the attention on all the considered variables involved in the experimentation, some considerations could be done. From an environmental point of view, only the strictly air-related variables (RH, VOC, CO2) are the most relevant descriptors of the comfort conditions, while the other thermal variables have a smaller impact. On the contrary, among biometric variables, skin temperature (Temp) is the relevant variable besides User in this specific study.

## 4. Conclusions

All previous considered works have not analyzed the correlation among environmental and biometric data using an ML approach in indoor space where greenery solutions are located. To overcome this limitation, the proposed approach describes a campaign investigation where the influences on both environmental and biometric parameters of participant of four different plant configuration are analyzed using wearable devices in addition to an environmental monitoring system.

Several results are carried out by the presented research.

The questions proposed in the previous Section 2 will be answered based on the presented results.

Research question 1: *What are the main environmental variables and models useful to accurately classify the adopted plant configurations*?

The evaluation of the comfort level of an indoor environment, according to its intended use, is usually carried out considering the IEQ assessment through a holistic and integrated study of different environmental aspects.

The presence of the living wall represents a forcing factor of some specific environmental variables concerning the sphere of thermal comfort among the others.

De facto, the oversized design of the adopted living wall with respect to the specific needs of the environment of the ZEB laboratory is, for example, a forcing agent with sensible effects on the degree of indoor relative humidity. However, this oversized design is in response to the study conducted on the green system which, as stated in the introduction, is wider than the one in object, and consequently essential.

The presence of the irrigation system, the specific lighting system for eight hours a day able to provide the most appropriate wavelength to the plants for proper growth and the evapotranspiration phenomena, are, in this specific case, forcing with significant effects on some variables, as they are effective in altering indoor microclimatic conditions.

Concretely, the indoor relative humidity degree undergoes a significant increase of up to 80% in the case of the presence of the living wall and air exchange systems being turned off (configuration 3) due to irrigation and evapotranspiration phenomenon.

All these considerations involve how, as reported in the Section 3.2.1, the RH feature has the most relevant impact when defining the plant configurations. Among the other environmental variables, in this specific case, the thermo-hygrometric variables (VA, TA, TA, TRA) have very little importance. As expected, a variable LX does not have a relevant weight in defining the adopted configuration. Among the air-quality-related features, VOC has an impact which is most relevant if compared with CO2.

Research question 2: *Are the biometric data useful to classify the adopted plant configurations? If so, which features are the most important*?

According to the bibliographic review, the biometric parameters that describe the physiological response of users to the indoor environment and its forcing agents can be influenced and altered according to the indoor environmental boundary conditions.

The studies analyzed have shown how biometric parameters can be influenced.

In the study, the biometric parameters and the answers to the users’ questionnaires were directly influenced by the presence of the living wall because, as highlighted above, it represents a forcing of the indoor microclimatic conditions.

Too-high values of relative humidity in the indoor environment, induced by the presence of the living wall and by the equipment suitable for its proper functioning, cause a variation in the temperature of the skin which is different for the considered users. That is why, among the biometric data, Temp and User have a relevant impact.

Research question 3: *How does combining environmental and biometric data could affect the accuracy of the model*?

Therefore, the application of the model cannot disregard the verification of how it behaves in the assessment of both biometric and physical indoor parameters in a combined manner. First, it can highlight how it is possible to replace, with good accuracy, the User values with a selected set of environmental and biometric data, thus overpassing the use of a categorical label.

In addition, it is possible to point out how, with the dominant effect recorded by the *RH* feature, in this specific case, the biometric data have a limited impact, except for the Temp data, which is more important than *CO_2_* in contributing to definition of the target values.

Beyond the answers to the questions, in the proposed research study, an index related to green elements viewing (GVF index) has been introduced to indicate the fraction of green area which occupies the surface of a hemisphere and could represent an interesting variable for deepening the study of green elements comfort impact in indoor spaces.

By a building operation point of view, specific environmental parameters are deeply influenced by the adopted plant configuration that also have an effect on the monitored biometric data. In particular, the variables analysis shows how the different aspects of internal comfort (thermal, air quality, lighting, acoustic) should be analyzed in an all-inclusive way due to the relationships that engage each other.

The ML approach used in the paper allows to characterize users by considering the selected features. This offers the opportunity to create a sort of “user archetypes”, implementable on building design in order to optimize building features.

Among the different considered ML techniques, the XGBoost-based model records the best performance in terms of target value identification.

The structured database can be used to define new a possible relation among monitored data and users’ feedback about their personal IEQ perception and this is a possible future improvement to the proposed work.

However, to maximize the replicability of this approach, some limitations that emerged during experimentation must be overcome.

Firstly, to maximize the potential of this approach, a new promising feature selection method can be considered in future development [59].

The experimentation has been carried out in a laboratory, with environmental variables which are not representative, in certain configurations, of a real working environment: in this context, it is difficult to scale the results to a real case study. For this reason, this first approach can be replicated considering a wider set of application in real case studies considering a greater variability in adopted greenery solution.

## Figures and Tables

**Table 1:** Main features of the reference literature and proposed study.

Reference Study	Test Environment	Greenery Element	Environmental Data	Biometric Data	Data Processing
[2]	Classroom	Real plants	Monitored	Not monitored	Statistical
[4]	Offices	Real plants	Monitored	Not monitored	Statistical
[8]	University	Living wall	Monitored	Not monitored	Statistical
[9]	University	Active Living Wall	Monitored	Not monitored	Statistical
[12]	University	Real plants and photos	Not monitored	Monitored	Statistical
[13]	Experimental room	Real plants	Not monitored	Monitored	Statistical
[20]	Hospital	Photos on monitor	Not monitored	Monitored	Statistical
[21]	Hospital	Photos	Not monitored	Not monitored	Statistical
[22]	Hospital	Real plants	Not monitored	Monitored	Statistical
[23]	Office	Real plants	Not monitored	Monitored	Statistical
[24]	School	Real plants	Not monitored	Monitored	Statistical
[25]	Office	Photos	Not monitored	Monitored	Statistical
[26]	School	Living wall	Not monitored	Not monitored	Statistical
[27]	Office	Real plants and Virtual Reality	Not monitored	Monitored	Statistical
Current study	Office lab	Living wall	Monitored	Monitored (IoT)	Machine Learning

**Table 2:** Characteristics of sensors installed in the test cell.

Sensor (Figure 2a)	Position (Figure 2a)	Variable	U.M.	Measure Range	Accuracy
TRH	In front of P1 and P2	Relative Humidity	[%]	0 ÷ 100%	±2 %
		Air Temperature	[°C]	−40 ÷ +60 °C	±0.1 °C
TRA	Near P1 and P2	Radiant Temperature (derived)	[°C]	−40 ÷ +60 °C	±0.1 °C
ANM	Left to P1 and P2	Air Velocity	[m/s]	0 ÷ 5 m/s	±0.02 m/s
		Air Temperature	[°C]	−20 ÷ +80 °C	±0.3 °C
LXL	In front of P1 and P2	Illuminance	[lx]		
CO2	Behind P1 and P2	CO2 concentration	[ppm]	0-5000 ppm	±50 ppm
VOC	Behind P1 and P2	Volatile Organic Compounds	[%]	0 ÷ 100 % of VOC	±20 %
PPG sensor	Smart wearable	Heat Recovery (HR) (derived)	[bpm]	-	-
EDA sensor	Smart wearable	EDA	[μS]	0.01 ÷ 100 µS	-
Skin temperature sensor	Smart wearable	Tskin	[°C]	−40 ÷ +85 °C	-
3-axes accelerometer	Smart wearable	Accelerations	[g]	±2 g	-

**Table 3:** Questions of Web-based survey.

Question ID	Questions	Answer Options
Q1	How do you evaluate the performance of your work?	1 (tiring) to 5 (untiring)
Q2	How do you assess thermo-hygrometric wellness on average?	1 (very unsatisfactory) to 5 (very satisfactory)
Q3	How do you assess the air quality on average?	1 (very unsatisfactory) to 5 (very satisfactory)
Q4	How do you assess the lighting quality on average?	1 (very unsatisfactory) to 5 (very satisfactory)

**Table 4:** Considered plant configurations and related setting.

Plant Configuration	Setting
1	Living wall absent HR OFF
2	Living wall absent HR ON
3	Living wall present HR OFF
4	Living wall present HR ON

**Table 5:** Dataset attributes and description.

ID	Label	Number	Type	Description
0	ID	5692	non-null int64	ID progressive
1	Data&Time	5692	non-null datetime64	Date and time
2	VA	5692	non-null float64	Air velocity [m/s] measured close to the workstations P1 and P2
3	TA	5692	non-null float64	Air temperature [°C] measured by the anemometer close to the workstations P1 and P2
4	AT	5692	non-null float64	Air temperature [°C] measured by the thermo-hygrometer close to the workstations P1 and P2
5	RH	5692	non-null float64	Relative humidity [%] measured by the thermo-hygrometer close to the workstations P1 and P2
6	TRA	5692	non-null float64	Radiant temperature [°C] measured by the globe thermometer close to the workstations P1 and P2
7	LX	5692	non-null float64	Illuminance [lx] measured by the luxmeter close to the workstations P1 and P2
8	CO2	5692	non-null int64	CO_2_ indoor concentration [ppm] close to the workstations P1 and P2
9	VOC	5692	non-null int64	VOCs [%] close to the workstations P1 and P2
10	EDA	5692	non-null float64	ElectroDermal Activity [μS]
11	AccelX	5692	non-null float64	Acceleration along the X axis [g]
12	AccelY	5692	non-null float64	Acceleration along the Y axis [g]
13	AccelZ	5692	non-null float64	Acceleration along the Z axis [g]
14	Temp	5692	non-null float64	Skin temperature [°C]
15	motion	5692	non-null float64	Root mean squared 3 axis acceleration [37,38]
16	HR	5692	non-null int64	Heart rate [bpm]
17	Q1	5692	non-null int64	Question 1 (see Table 3 for more details)
18	Q2	5692	non-null int64	Question 2 (see Table 3 for more details)
19	Q3	5692	non-null int64	Question 3 (see Table 3 for more details)
20	Q4	5692	non-null int64	Question 4 (see Table 3 for more details)
21	IEQ_avg	5692	non-null float64	IEQ as a weighted average of previous scores
22	User	5692	non-null float64	Number of the user
23	P1/P2	5692	non-null object	P1/P2 workstation
24	M/A	5692	non-null object	Morning/Afternoon
25	Plant_Config.	5692	non-null int64	Configuration as reported in Table 4

**Table 6:** Average accuracy—selected environmental data for plant configuration identification. With *: average accuracy defined considering the tuning of hyperparameters.

Algorithm	Average Accuracy		Standard Deviation	
	25 = *ƒ* [5,8,9]	25 = *ƒ* [5,9]	25 = *ƒ* [5,8,9]	25 = *ƒ* [5,9]
LR	0.988*	0.988*	0.006*	0.005*
LDA	0.969	0.634	0.008	0.028
KNN	0.982*	0.994*	0.005	0.005*
CART	0.997	0.974*	0.002	0.008*
ETC	0.993	0.875	0.006	0.015
NB	0.984	0.621	0.006	0.027
SVM	0.977*	0.986*	0.008*	0.007*
RF	0.998*	0.994*	0.002*	0.005*
XGBoost	0.998*	0.995*	0.002*	0.004*

**Table 7:** Hyperparameters tuning range.

Algorithms	Hyperparameters	Range
LR	Solver	[‘newton-cg’, ‘lbfgs’, ‘liblinear’]
	Penalty	[‘l1′, ‘l2′, ‘elasticnet’, ‘none’]
	C_value	[100, 10, 1.0, 0.1, 0.01]
KNN	Leaf_size	range(1,10,2)
	n_neighbors	range(1,30,5)
	p_value	[1,2]
CART	Max_depth	range(1,50,4)
	Min_samples_leaf	[i/10.0 for i in range(1,6)]
	Max_features	[i/10.0 for i in range(1,11)]
ETC	Max_depth	range(1,50,4)
	Min_samples_leaf	[i/10.0 for i in range(1,6)]
	Max_features	[i/10.0 for i in range(1,11)]
SVM	Kernel	[‘poly’, ‘rbf’, ‘sigmoid’]
	C_value	[50, 10, 1.0, 0.1, 0.01]
RF	n_estimators	range(1,22,2)
XGBoost	Max_depth	range(3,10,2)
	Min_child_weight	range(1,6,2)
	Gamma	[i/10.0 for i in range(0,5)]

**Table 8:** Validation—selected environmental data for plant configuration identification.

	Plant Config.	Precision	Recall	F1-Score	Support
RF, 25 = *ƒ* [5,8,9]	1	0.98	0.87	0.92	119
	2	0.95	0.99	0.97	290
	3	1.00	1.00	1.00	330
	4	1.00	1.00	1.00	400
XGBoost, 25 = *ƒ* [5,8,9]	1	1.00	1.00	1.00	119
	2	0.99	0.99	1.00	290
	3	1.00	0.99	1.00	330
	4	0.98	1.00	1.00	400
	1	1.00	0.95	0.97	119
XGBoost, 25 = *ƒ* [5,9]	2	0.98	1.00	0.99	290
	3	1.00	1.00	1.00	330
	4	0.99	1.00	0.99	400

**Table 9:** Average accuracy—selected biometric data for plant configuration identification. With *: average accuracy defined considering the tuning of hyperparameters.

Algorithm	Avgerage Accuracy		Standard Deviation	
	25 = *ƒ* [10,11,14,22]	25 = *ƒ* [14,22]	25 = *ƒ* [10,11,14,22]	25 = *ƒ* [14,22]
LR	0.500*	0.500*	0.015*	0.015*
LDA	0.363	0.363	0.024	0.023
KNN	0.871*	0.742*	0.011	0.019*
CART	0.855	0.626	0.011	0.016
ETC	0.848	0.632	0.024	0.024
NB	0.397	0.347	0.015	0.024
SVM	0.559*	0.495*	0.015*	0.014*
RF	0.891*	0.687*	0.011	0.020*
XGBoost	0.902*	0.743*	0.014	0.016*

**Table 10:** Validation—selected biometric data for plant configuration identification.

	Plant Config.	Precision	Recall	F1-Score	Support
XGBoost, 25 = *ƒ* [10,11,14,22]	1	0.94	0.92	0.93	119
	2	0.82	0.84	0.83	290
	3	0.92	0.91	0.91	330
	4	0.85	0.85	0.85	400
XGBoost, 25 = *ƒ* [14,22]	1	0.89	0.92	0.91	119
	2	0.70	0.68	0.69	290
	3	0.74	0.70	0.72	330
	4	0.70	0.73	0.71	400

**Table 11:** Average accuracy—selected environmental and biometric data for user identification. With *: average accuracy defined considering the tuning of hyperparameters.

Algorithm	Average Accuracy	Standard Deviation
LR	0.364*	0.018
LDA	0.217	0.021
KNN	0.963*	0.006*
CART	0.816	0.009
ETC	0.788	0.013
NB	0.321	0.030
SVM	0.251*	0.015*
RF	0.957*	0.009*
XGBoost	0.959*	0.008*

**Table 12:** Validation—selected environmental and biometric data for user identification.

	User	Precision	Recall	F1-Score	Support
XGBoost, 22 = *ƒ* [5,9,14]	1	0.99	0.97	0.98	106
	2	0.94	0.98	0.96	138
	3	0.92	0.95	0.93	83
	4	0.91	0.95	0.93	63
	5	0.96	0.93	0.94	137
	6	0.95	0.94	0.94	160
	7	0.98	0.91	0.94	159
	8	0.95	0.97	0.96	162
	9	0.93	0.96	0.95	131

**Table 13:** Average accuracy—selected environmental and biometric data for plant configuration identification. With *: average accuracy defined considering the tuning of hyperparameters.

Algorithm	Average Accuracy		Standard Deviation	
	25 = *ƒ* [5,8,9,10,11,14]	25 = *ƒ* [5,9,14]	25 = *ƒ* [5,8,9,10,11,14]	25 = *ƒ* [5,9,14]
LR	0.977*	0.952*	0.005*	0.008*
LDA	0.964	0.937	0.008	0.008
KNN	0.998*	0.986*	0.002*	0.005*
CART	0.984	0.983	0.008	0.007
ETC	0.978	0.977	0.005	0.007
NB	0.605	0.933	0.026	0.008
SVM	0.961*	0.967*	0.009	0.007*
RF	0.994*	0.986*	0.003	0.004*
XGBoost	0.998*	0.986*	0.002	0.004*

**Table 14:** Validation—selected environmental and biometric data for plant configuration identification.

	Plant Config.	Precision	Recall	F1-Score	Support
XGBoost, 25 = *ƒ* [5,8,9,10,11,14]	1	1.00	1.00	1.00	119
	2	1.00	1.00	1.00	290
	3	1.00	1.00	1.00	330
	4	1.00	1.00	1.00	400
XGBoost, 25 = *ƒ* [5,9,14]	1	0.98	1.00	0.99	119
	2	1.00	0.99	1.00	290
	3	0.98	0.99	0.98	330
	4	1.00	0.99	0.99	400
	1	0.98	1.00	0.99	119
RF, 25 = *ƒ* [5,9,14]	2	1.00	0.99	1.00	290
	3	0.98	0.98	0.98	330
	4	0.99	0.98	0.99	400
